# Strategies to improve reference databases for soil microbiomes

**DOI:** 10.1038/ismej.2016.168

**Published:** 2016-12-09

**Authors:** Jinlyung Choi, Fan Yang, Ramunas Stepanauskas, Erick Cardenas, Aaron Garoutte, Ryan Williams, Jared Flater, James M Tiedje, Kirsten S Hofmockel, Brian Gelder, Adina Howe

**Affiliations:** 1 Department of Agricultural and Biosystems Engineering, Iowa State University, Ames, IA, USA; 2 Bigelow Laboratory for Ocean Sciences, East Boothbay, ME, USA; 3 Department of Microbiology & Immunology, University of British Columbia, Vancouver, BC, Canada; 4 Center for Microbial Ecology, Michigan State University, East Lansing, MI, USA; 5 Environmental Molecular Sciences Laboratory, Pacific Northwest National Laboratory, Richland, WA, USA; 6 Department of Ecology, Evolution and Organismal Biology, Iowa State University, Ames, IA, USA

## Introduction

Microbial populations in the soil are critical in our lives. The soil microbiome helps to grow our food, nourishing and protecting plants, while also providing important ecological services such as erosion protection, water filtration and climate regulation. We are increasingly aware of the tremendous microbial diversity that has a role in soil heath; yet, despite significant efforts to isolate microbes from the soil, we have accessed only a small fraction of its biodiversity. Even with novel cell isolation techniques, <1–50% of soil species have been cultivated (Janssen *et al.*, 2002; Van Pham and Kim, 2012). Metagenomic sequencing has accelerated our access to environmental microbes, allowing us to characterize soil communities without the need to first cultivate isolates. However, our ability to annotate and characterize the retrieved genes is dependent on the availability of informative reference gene or genome databases.

The current genomic databases are not representative of soil microbiomes. Contributions to the existing databases have largely originated from human health and biotechnology research efforts and can mislead annotations of genes originating from soil microbiomes (for example, annotations that are clearly not compatible with life in soil). Soil microbiologists are not the first to face the problem of a limited reference database. The NIH Human Microbiome Project (HMP) recognized the critical need for a well-curated reference genome dataset and developed a reference catalog of 3000 genomes that were isolated and sequenced from human-associated microbial populations (Huttenhower *et al.*, 2012). This publicly available reference set of microbial isolates and their genomic sequences aids in the analysis of human microbiome sequencing data (Wu *et al.*, 2009; Segata *et al.*, 2012) and also provides strains for which isolatese (both culture collections and nucleic acids) are available as resources for experiments.

Our increasing awareness of the links between microbial communities and soil health has resulted in significant investments in using sequence-based approaches to understand the soil microbiome. The Earth Microbiome Project (www.earthmicrobiome.org) alone is characterizing 200 000 samples from researchers all over the world. Despite increasing volumes of soil sequencing datasets, we currently lack soil-specific genomic resources to inform these studies. To fill this need, we have curated *RefSoil* (See Supplementary Methods) from the genomic data that originates from cultured representatives originating from soil. RefSoil (both its genomes and associated strain isolates) provides a soil-specific framework with which to annotate and understand soil sequencing projects. Additionally, its curation is the first step in identifying strains that are currently gaps in our understanding of soil microbiology, allowing us to strategically target them for cultivation and characterization. In this perspective, we introduce RefSoil and highlight several examples of its applications that would benefit diverse users.

## RefSoil: a soil microbiome database

We have curated a reference database of sequenced genomes of organisms from the soil, naming it *RefSoil* (See Supplementary Methods). The RefSoil genomes are a subset of NCBI's database of sequenced genomes, RefSeq (release 74), and have been manually screened to include only organisms that have previously been associated with soils. RefSoil contains a total 922 genomes, 888 bacteria and 34 archaea (Supplementary Table 1). While sharing similar dominant organisms to the RefSeq database (for example, Proteobacteria, Firmicutes and Actinobacteria), RefSoil contains higher proportions of Armatimonadetes, Germmatimonadetes, Thermodesulfobacteria, Acidobacteria, Nitrospirae and Chloroflexi, suggesting that these phyla may be enriched in the soil or under-represented in RefSeq. A total of 11 RefSeq-associated phyla are not included in RefSoil and these phyla are most likely absent or difficult to cultivate in soil environments (Supplementary Figure 1).

RefSoil can be used to define a representative framework that can provide insight into potential soil functions and genes, and phyla that are associated with encoding functions. We observe that genes related to microbial growth and reproduction (for example, DNA, RNA and protein metabolism) are associated with diverse RefSoil phyla; in contrast, key functions related to metabolism of aromatic compounds and iron metabolism are enriched in Proteobacteria and Actinobacteria. Similarly, dormancy and sporulation genes are enriched in Firmicutes (Supplementary Figure 2, Supplementary Tables 2 and 3). Many of the broader functions encoded by RefSoil genes are unsurprising (for example, photosynthesis in Cyanobacteria), but as a collective framework, RefSoil genomes and their associated isolated strains can allow us to look deeper into soil functions. Specifically, understanding the functions encoded by specific soil membership can guide the selection and design of representative mock communities for soil processes. For example, an experimental community of isolates known for participating in nitrogen cycling could include RefSoil strains related to that associated with assimilatory nitrate reductase nitric and nitrous oxide reductase ammonia monooxygenase and nitrogen fixation (selected from Supplementary Figure 2). Another potential opportunity for RefSoil is to provide context that can help improve functional annotation of genomes. The large majority of genes in previously published soil metagenomes (65–90%) cannot be annotated against known genes (Delmont *et al.*, 2012; Fierer *et al.*, 2012). By comparing uncharacterized RefSoil genes shared between multiple strains, representative strains could be selected for experimental characterization that could lead to protein annotation. These specific examples highlight the value of RefSoil to broad researchers, both experimental and computational, to improve our understanding of soil function. Going forward, integrating computational and experimental strategies will be significant to provide the most insight into this complex system.

## How representative are our existing references in natural soils?

While we are able to glimpse into soil microbial ecology through RefSoil's genomes, its ability to inform natural soils depends on the representation of laboratory isolates in our soils. There are now datasets to assess global soil microbiomes through efforts like the Earth Microbiome Project (EMP) (Gilbert *et al.*, 2014; Rideout *et al.*, 2014), which have collected a total of 3035 soil samples and sequenced their associated 16S rRNA gene amplicons. Clustering at 97% sequence similarity, these EMP OTUs represent 2158 unique taxonomic assignments (See Supplementary Methods), with varying abundances estimated in each soil sample (for example, total count of amplicons). We observed that the majority of these OTUs are rare (for example, only observed in a few samples) with 76% of OTUs observed in <10 soil samples, and 1% of OTUs representing 81% of total sequence abundance in EMP.

**Figure 1:**
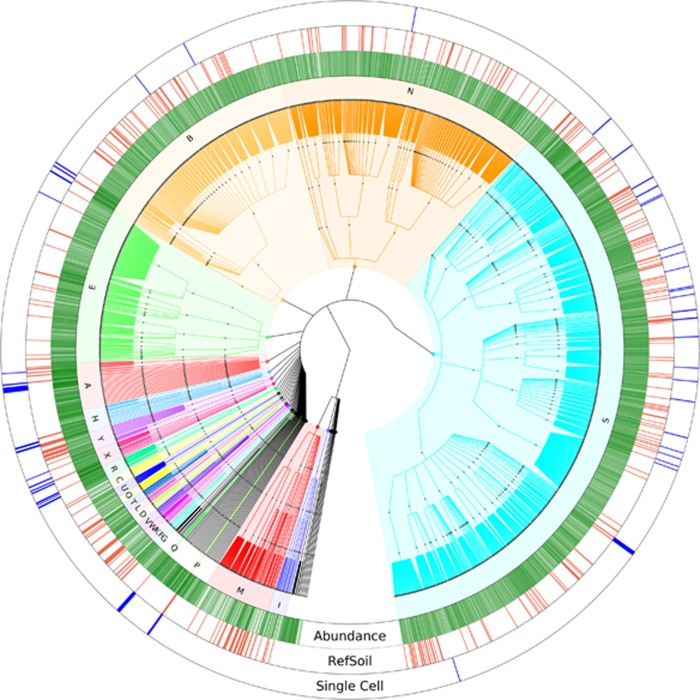
Phylogenetic tree of EMP OTUs clustered by taxonomy. Ring I (green) represents the cumulative log-scaled abundance of OTUs in EMP soil samples. Ring II (red) represents EMP OTUs that share >97% gene similarity (to RefSoil 16S rRNA genes; ring III (blue) indicates that these 16S rRNA genes shared similarity to sorted cells that were selected for single-cell genomics. A: Acidobacteria, B: Actinobacteria, C: Aquificae, D: Armatimonadetes, E: Bacteroidetes, F: Chlamydiae, G: Chlorobi, H: Chloroflexi, I: Crenarchaeota, K: Deferribacteres, L: Deinococcus-Thermus, M: Euryarchaeota, N: Firmicutes, O: Fusobacteria, P: Gemmatimonadetes, Q: Nitrospirae, R: Planctomycetes, S: Proteobacteria, T: Spirochaetes, U: Synergistetes, V: Tenericutes, W: Thermotogae, X: Verrucomicrobia, Y: Cyanobacteria/Chloroplast.

To evaluate the presence of RefSoil genomes in soil samples, EMP 16S rRNA gene amplicons and RefSoil 16S rRNA genes were compared, requiring an alignment with >97% similarity, a minimum alignment of 72 bp, and *E*-value ≤1e-5. Using these criteria, a total of 53 538 EMP OTUs shared similarity with RefSoil 16S rRNA genes. These OTUs represent a meager 1.4% of all EMP diversity (unique OTUs) or 10.2% of all EMP amplicon sequences. Overall, we observe that 99% (2 442 432 of 2 476 795) of observed EMP amplicons do not share >97% similarity to RefSoil genes, suggesting that EMP soil samples contain much higher diversity than represented within RefSoil (Figure 1) and highlights the poor representation of our current reference genomes. Notably, Firmicutes are observed frequently in the RefSoil database (Supplementary Figure 3) but are not observed to be highly abundant in soil environments (5.7% of all EMP amplicons). Firmicutes have been well-studied as pathogens, (Rupnik *et al.*, 2009; Buffie and Pamer, 2013), likely resulting in their biased representation in our databases and consequently also biased annotations in soil studies. A key advantage to the development of the RefSoil database is the opportunity to identify these biases and to ensure increasingly representative targets for future curation efforts. In annotating soil metagenomes with public databases, organisms and genes that are not associated with soils can consistently be identified; for example, in an Iowa corn metagenome annotated by the MG-RAST database, we identified both sea anemone and corals (MG-RAST ID: 4504797.3). While the broad public gene databases contain significantly larger numbers of genes compared with RefSoil, one must cautiously leverage them so as not to interpret misleading results.

## Recommended direction forward for soil references

By comparing RefSoil with the EMP datasets, we are able to identify genome targets where we lack available reference genomes and whose genes have been observed to be highly abundant in soils (Figure 1, green bars). Using these two criteria, we have generated a ‘most wanted OTUs' list for expanding RefSoil to increase its representation of soil biodiversity (Table 1). Candidate OTU targets were ranked based on their observed frequency in all EMP samples and abundance in EMP amplicons (Top 100 shown in Supplementary Tables 4 and 5). We observed that OTUs sharing similarity to Verrucomicrobia (8 OTUs) and Acidobacteria (6 OTUs) were among the most abundant and frequently observed EMP OTUs that are not currently represented in RefSoil (Table 1). Both these phyla are well known for their difficulty to isolate in laboratory conditions. Acidobacteria, for example, is known to be slow growing (Nunes da Rocha *et al.*, 2009) despite its abundance in soil (33% of EMP amplicons by abundance). Verrucomicrobia are also fastidious (Fierer *et al.*, 2013) and highly abundant in soils (12.5% in EMP) but not well represented in RefSoil (2 of 888 bacterial genomes). Despite their absence from cultivated isolates, both Acidobacteria and Verrucomicrobia have been observed to be critical for nutrient cycling in soils (Nunes da Rocha *et al.*, 2009; Fierer *et al.*, 2013). As we continue to isolate and sequence genomes from soils, the 16S rRNA sequences of these and other most-wanted OTUs can help prioritize efforts among isolates, and soil samples where these OTUs are observed may aid in cultivation efforts. By obtaining genome references for the top most wanted organisms identified in this effort (Table 1), we could expand RefSoil's representation of EMP soils by 1.6-fold by abundance. Using RefSoil and EMP, microbiologists could strategically target isolate characterization to fill in gaps in our knowledge base and provide novel information for understanding soil microbiology.

## Soil single cell genomics

Sequencing-based approaches provide another exciting alternative to accessing the genomes of soil organisms without cultivation. Previous efforts have used assembly of genomes from metagenomes (Hultman *et al.*, 2015) and single-cell genomics (Stepanauskas, 2012; Gawad *et al.*, 2016) to obtain genomic blueprints of yet uncultured microbial groups. To evaluate the effectiveness of single cell genomics on soil communities, we performed a pilot-scale experiment on a residential garden soil in Maine, USA. The 16S rRNA gene was successfully recovered from 109 of the 317 single amplified genomes (SAGs). This 34% 16S rRNA gene recovery rate is comparable to single cell genomics studies in marine, freshwater and other environments (Swan *et al.*, 2011; Rinke *et al.*, 2013). The 16S rRNA genes of these 14 SAGs, belonging to Proteobacteria, Actinobacteria, Nitrospirae, Verrucomicrobia, Planctomycetes, Acidobacteria and Chloroflexi were selected based on their lack of representation within RefSoil and observed abundances in EMP OTUs (Figure 1). Genomic sequencing of those SAGs resulted in a cumulative assembly of 23 Mbp (Table 2, Supplementary Table 6). We estimate the equivalent EMP-abundance represented by these SAGs to be <1% of total EMP OTU abundances. While these abundances are very low, they are comparable to the average relative abundance of OTUs observed in EMP. If all sequenced SAG genomes were added to RefSoil, its representation of EMP OTUs would increase by 7% by abundance.

Going forward, novel isolation and culturing techniques complemented by emerging sequencing technologies will provide us access to previously difficult to grow bacteria. In particular, single-cell genomics hold great promise to provide genomic characterization of lineages that are difficult to culture (Stepanauskas, 2012; Gawad *et al.*, 2016). In our pilot experiment, we demonstrate, for the first time, that single-cell genomics is applicable on soil samples and is well suited to recover the genomic information from abundant, but yet uncultured taxonomic groups. The 14 sequenced SAGs have significantly increased the extent to which RefSoil represents the predominant soil lineages from a single sample. Much larger single-cell genomics projects are feasible and have been employed in prior studies of other environments(Rinke *et al.*, 2013; Kashtan *et al.*, 2014). The continued, rapid improvements in this technology are likely to lead to further scalability, offering a practical means to fill the existing gaps in the RefSoil database and biodiversity more broadly.

## RefSoil applications beyond soil sequence annotation

To demonstrate another application of RefSoil, we assessed the distribution of RefSoil genomes in various soil types. We used the soil taxonomy developed by the United States Department of Agriculture (USDA) and the National Resources Conservation Service, which separates soils into 12 orders based on their physical, chemical or biological properties (See Supplementary Methods). Despite the availability of this classification, it is rarely incorporated into soil microbiome surveys. Using RefSoil and estimated abundances from similar EMP OTUs, we evaluated the distribution of soil isolates in various soil orders. We obtained the GPS data and corresponding soil classification of EMP soil samples originating from the United States. Within these EMP samples, the most represented soil orders included Mollisols (58%, grassland fertile soils) and Alfisols (37%, fertile soils typically under forest vegetation) (Supplementary Table 7). Mollisols, Alfisols and Vertisols (soils with high clay content with pronounced changes in moisture) were associated with the most RefSoil representatives, while Gelisols (cold climate soils), Ultisols (soils with low cation exchange), and sand/rock/ice contained very few RefSoil representatives (Supplementary Figure 4 and Supplementary Table 7). These results are consistent with previous observations that microbial community composition varies depending on soil environments (Fierer *et al.*, 2012). Further, we observe that soil studies and our references are heavily biased towards agricultural or productive soils, and there is much we do not know about understudied soils such as permafrost and desert soils.

## Conclusion

Advances in sequencing techniques for utilizing culture-independent approaches have created tremendous opportunities for understanding soil microbiology and its impact on soil health, stability and management. Currently, our ability to convert this growing sequencing data to information is severely limited and skewed by the representation of current genome reference databases. Here, we provide an initial effort in the curation of a soil-specific community genomic resource and identify currently underrepresented soil phyla and their genomes. Given that the large majority of soil metagenomes cannot currently be annotated by publicly available references, the curation and expansion of environment-specific references is a feasible first step towards improving annotation. RefSoil provides informed selection of future genome targets, allowing us to more efficiently fill in knowledge gaps. As soil reference genomes improve, our ability to leverage other omic-based approaches will improve. Another important opportunity going forward with this resource is the integration of other genomic resources to continue to improve soil-specific resources. In this particular effort, RefSeq and EMP datasets were combined with single-cell genomics to increase soil genome references. Additionally, efforts to integrate and compare other environment-specific databases (for example, HMP reference genomes or the broader RefSeq genomes) and the thousands of publicly available metagenomes could help us better understand the role of microbiomes on our lives.

## Figures and Tables

**Table 1:** RefSoil's most wanted OTUs

*OTU ID in (Rideout et al., 2014)*	*Closest match in RDP classifer*		*RDP classifier similarity score*	*Abundance (total amplicons)*	*Number of samples*
	*Phylum*	*Class*			
4457032	Verrucomicrobia	Spartobacteria	1	8 007 453	2312
4471583	Verrucomicrobia	Spartobacteria	0.92	2 937 242	1935
101868	Verrucomicrobia	Spartobacteria	1	1 828 706	2151
559213	Firmicutes	Bacilli	1	1 606 757	2410
1105039	Verrucomicrobia	Spartobacteria	1	1 295 847	2102
807954	Bacteroidetes	Sphingobacteriia	0.98	875 988	2546
4342972	Verrucomicrobia	Spartobacteria	0.97	750 557	2386
4423681	Gemmatimonadetes	Gemmatimonadetes	0.25	689 209	1954
1109646	Verrucomicrobia	Spartobacteria	1	554 748	2012
610188	Acidobacteria	Acidobacteria_Gp6	0.99	553 476	2694
4373456	Acidobacteria	Acidobacteria_Gp4	1	397 255	2150
4341176	Verrucomicrobia	Subdivision3	0.99	383 900	2098
720217	Proteobacteria	Deltaproteobacteria	0.74	345 621	2073
4314933	Acidobacteria	Acidobacteria_Gp1	0.8	333 428	1974
205391	Acidobacteria	Acidobacteria_Gp3	0.87	327 162	2190
4378940	Acidobacteria	Acidobacteria_Gp6	1	310 421	2499
946250	Verrucomicrobia	Spartobacteria	1	300 544	2386
3122801	Acidobacteria	Acidobacteria_Gp1	0.97	273 493	2107
4450676	Proteobacteria	Alphaproteobacteria	1	255 424	1963
206514	Proteobacteria	Alphaproteobacteria	0.92	207 460	2075
4463040	Firmicutes	Clostridia	0.94	204 483	2096

RefSoil's most wanted OTUs based on observed frequency and abundance in EMP soil samples. Taxonomy for OTUs are assigned by RDP classifier(Wang *et al.*, 2007). *:(Rideout *et al.*, 2014).

**Table 2:** Single-cell amplified genomes

*NCBI ID*	*Phylum*	*Class*	*EMP ID in (Rideout et al., 2014)*	*Abundance*	*Relative abundance (%)*
LSTF00000000	Proteobacteria	β-proteobacteria	New.52.CleanUp.ReferenceOTU25474	81 630	2.13E-02
LSTI00000000	Actinobacteria	Thermoleophilia	551344	35 500	9.27E-03
LSTA00000000	Verrucomicrobia	Spartobacteria	New.54.CleanUp.ReferenceOTU60738	5076	1.33E-03
LSTC00000000	Nitrospirae	Nitrospira	New.22.CleanUp.ReferenceOTU3952	4337	1.13E-03
LSTH00000000	Planctomycetes	Planctomycetia	521158	4161	1.09E-03
LSSZ00000000	Planctomycetes	Planctomycetia	New.5.CleanUp.ReferenceOTU188938	2434	6.36E-04
LSTE00000000	Proteobacteria	γ-proteobacteria	NA	NA	NA
LSTB00000000	Proteobacteria	α-proteobacteria	New.52.CleanUp.ReferenceOTU4588	309	8.07E-05
LSSY00000000	Actinobacteria	Thermoleophilia	904196	103	2.69E-05
LSTG00000000	Verrucomicrobia	Opitutae	New.5.CleanUp.ReferenceOTU291124	69	1.80E-05
LSTD00000000	Planctomycetes	Planctomycetia	New.59.CleanUp.ReferenceOTU1223798	44	1.15E-05
LSTJ00000000	Verrucomicrobia	Verrucomicrobiae	New.7.CleanUp.ReferenceOTU84651	11	2.87E-06
LSTK00000000	Acidobacteria	Acidobacteriia	New.59.CleanUp.ReferenceOTU999655	4	1.04E-06
LSSX00000000	Chloroflexi	TK17	New.59.CleanUp.ReferenceOTU481621	2	5.22E-07

Taxonomic classification of single-cell amplified genomes and the abundance of the most similar EMP OTU. *: (Rideout *et al.*, 2014).
