# Supplementary material for: Strategies to improve reference databases for soil microbiomes
Source: ISME J. 2016 Dec 9;11(4):829–34. doi: 10.1038/ismej.2016.168 (PMC5364351; doi:10.1038/ismej.2016.168)
Supplement: Supplementary Information [file ismej2016168x1.docx]

**Supplementary Methods**

**Curation of RefSoil**

Genomes were obtained from the Genomes OnLine Database (GOLD, <https://gold.jgi.doe.gov>, October 9th, 2014); the GOLD database was chosen because of the availability of metadata, particularly environmental origin, related to genome sequences. These genomes were further selected based on soil-association with the following criteria: (1) Within the GOLD database, organism information and organism metadata (known habitats, ecosystem category, and ecosystem type) was required to identify organism as originating in soil-associated categories. Organisms from marine and deep sea environments were excluded, though these organisms often were identified as soil-associated. Additionally, obligate host-associated pathogens and extremophiles were excluded, as these organisms are unlikely to be present in the absence of their host or in representative soil samples. We considered these organisms to often be under very strong selective pressures that can lead to reduced genomes or high rates of recombination that are difficult to assess soil-specific trends. (2) For organisms that lacked appropriate metadata in GOLD, a Google Custom Search was used to query all available websites for the organism name and soil-related terms (rhizosphere, soil, sand, mud, or nodule). Genomes with an association with at least one webpage containing search query phrases were included in RefSoil. This approach was tested with known soil-associated organisms, verifying that it would reliably predict soil association for these organisms. Within resulting genomes, duplicated chromosomes and strains were removed. If multiple genome accession numbers were associated with a single strain, the most recent genome sequence was chosen. NCBI Genbank annotation files associated with each strain were obtained on 2/18/16. The genomes contained within RefSoil are provided in Supplementary Table 1.

**Characterization of organisms in RefSoil**

All RefSoil genome sequences were associated with their NCBI RefSeq accession ID and obtained from NCBI. Using NCBI Genbank CDS annotations, 16s rRNA gene sequences were identified. If multiple 16S rRNA gene sequences were present, the first sequence from the first chromosome (by NCBI index) was selected as representative for the genome. Three genomes lacked annotations of a 16S rRNA gene and a 16S rRNA gene HMM model was used and identified an additional 16S rRNA sequence(Guo, Cole, Zhang, Brown, & Tiedje, 2015). In total, 886 16S rRNA gene sequences were used to build a phylogenetic tree for bacterial genomes within RefSoil. In total, 886 16S rRNA gene sequences were used to build a phylogenetic tree for bacterial genomes within RefSoil. All bacterial 16S rRNA gene sequences were aligned using RDP’s bacterial model (Infernal 1.1.1 (Nawrocki & Eddy, 2013)), and a Maximum-likelihood phylogenetic tree was constructed based on the Jukes-Cantor model by Fasttree (Price, Dehal, & Arkin, 2010) and visualized with Graphlan (R 3.2.2, version 0.9.7) (Supplementary Figure 3). The scripts for these approaches are publicly available on https://github.com/germs-lab/ref_soil. RefSoil genomes were extracted from corresponding genomes contained within NCBI RefSeq (release 74). Annotations of taxonomy for RefSoil and RefSeq genes were obtained from NCBI (February 19, 2016) (Supplementary Figure 1).

We examined the functions represented by RefSoil genomes, annotating these genomes with the Rapid Annotation using Subsystem Technology (RAST, v 2.0(Aziz et al., 2008)) A total of 1,811,233 genes were annotated, with 78% of these genes classified into functional ontologies or subsystems (Supplementary Table 2). approximately 39.1% of them were assigned to multiple SEED subsystem level 1 categories. For the purpose of this study, we included all annotations at subsystem level 1 for each unique gene, which expanded the total gene counts to 2,619,643. The percent abundance of genes from each phylum in RefSoil database was also adjusted accordingly by including the abundance of genes that were assigned to multiple subsystem level 1 categories (Supplementary Figure 2 and Supplement Table 3). The number of phyla identified in each subsystem level 1 categories was summarized in Supplemental Table 3. For genes associated with each subsystem, we evaluated the phylogenetic origins of RefSoil genes and compared the phlya distribution of annotated genes with those within the cumulative RefSoil database (Supplementary Figure 2). If the proportion of genes associated with a phylum within a functional subsystem was greater than its representation in RefSoil, we considered the representation of the phyla *enriched* in this function. For example, we observed that in most subsystems (15 out of 26 subsystems), Proteobacteria-associated genes were enriched relative to their representation within RefSoil (56% of all RefSoil genes associated with Proteobacteria). Additionally, Actinobacteria, Crenarchaeota, and Proteobacteria genes were enriched in functions related to metabolism of aromatic compounds; Firmicutes and Proteobacteria genes were enriched in functions related to iron acquisition and metabolism; and function category dormancy and sporulation was enriched with Firmicutes genes only. These enrichments indicate that organisms from a small number of phyla may have advantages over other organisms in environments where these functions are important. (Supplementary Figure 2, Supplementary Table 3)

**Datasets used in this study**

A total of 15,481 amplicon datasets that form the EMP dataset were available to compare environmental soil amplicons to RefSoil (total 5,594,412 OTUs, clustered at 97%)(Rideout et al., 2014). Soil samples were selected based on their association with soil metadata resulting in a total of 2,476,795 OTUs from 3035 samples. A total of 2,158 unique taxonomy assignments were identified for EMP OTUs using the RDP Classifier (Wang, Garrity, Tiedje, & Cole, 2007) and used to construct a phylogenetic tree with the Graphlan package (R 3.2.2, version 0.9.7) (Figure 1). Abundances for each taxonomic assignment were calculated as the sum of abundance of OTUs associated with that taxonomy.

**Single cell genomics**

For single cell genomics, a soil sample was collected from 0-10 cm depth in a residential garden in Nobleboro, Maine (44° 5'48.10"N, 69°29'10.56"W) on May 5^th^, 2015. Approximately five grams of the sample were mixed with 30 mL sterile-filtered phosphate-saline buffer (PBS), vortexed for 30 s at maximum speed, and centrifuged for 30 s at 2,000 rpm. The obtained supernatant was diluted to below 10^5^ cell x mL^-1^ with PBS, pre-screened through a 40 μm mesh-size cell strainer (BD), and incubated with SYTO-9 DNA stain (5 μM; Invitrogen) for 10-60 min. The generation of single amplified genomes (SAGs) and their genomic sequencing were performed by the Bigelow Laboratory Single Cell Genomics Center (scgc.bigelow.org), as previously described(Stepanauskas et al., n.d.). SAGs representing the "most-wanted list" were selected based on 16S rRNA gene BLAST alignments with greater than 97% similarity over at least 72 bp.

**RefSoil genomes in various soil types**

Soil order for EMP samples located in the United States (1817 samples) was obtained based on GPS location associated with sample metadata(Soil Survay Staff 1999, 1999); locations were only considered valid if the coordinates entered were actually located in the United States (1627 samples). Valid GPS points were then located within a soil order by querying the USDA NRCS Global Soil Regions map (Reich, n.d.) (Accessed on January 26, 2016) for soil order plus a Rock/Sand/Ice category using ArcGIS 10.3.1. RefSoil representatives for EMP OTUs were determined by similarity as described above (Supplementary Table 7).

**Code and sequencing data availability**

The analysis code used to generate the results is available from <https://github.com/germs-lab/ref_soil>. All 14 single-cell sequencing data sets have been deposited at the NCBI. Sequencing data with NCBI accession identifier are listed in Table 6.

**References**

Aziz, R. K., Bartels, D., Best, A. A., DeJongh, M., Disz, T., Edwards, R. A., et al. (2008). The RAST Server: Rapid Annotations using Subsystems Technology. *BMC Genomics*, *9*(1), 75. http://doi.org/10.1186/1471-2164-9-75

Guo, J., Cole, J. R., Zhang, Q., Brown, C. T., & Tiedje, J. M. (2015). Microbial Community Analysis with Ribosomal Gene Fragments from Shotgun Metagenomes. *Applied and Environmental Microbiology*, *82*(1), 157–166. http://doi.org/10.1128/AEM.02772-15

Nawrocki, E. P., & Eddy, S. R. (2013). Infernal 1.1: 100-fold faster RNA homology searches. *Bioinformatics*, *29*(22), 2933–2935. http://doi.org/10.1093/bioinformatics/btt509

Price, M. N., Dehal, P. S., & Arkin, A. P. (2010). FastTree 2 – Approximately Maximum-Likelihood Trees for Large Alignments. *PLoS ONE*, *5*(3), e9490. http://doi.org/10.1371/journal.pone.0009490

Reich, P. (n.d.). Global soil suborders. Retrieved from http://www.nrcs.usda.gov/wps/portal/nrcs/detail/soils/use/?cid=nrcs142p2_054013

Rideout, J. R., He, Y., Navas-Molina, J. A., Walters, W. A., Ursell, L. K., Gibbons, S. M., et al. (2014). Subsampled open-reference clustering creates consistent, comprehensive OTU definitions and scales to billions of sequences. *PeerJ*, *2*(5), e545–25. http://doi.org/10.7717/peerj.545

Soil Survay Staff 1999. (1999). Soil Taxonomy: A basic system of soil classification for making and interpreting soil surveys (2nd ed.). Natural Resources Conservation Service: U.S. Department of Agriculture Handbook 436.

Stepanauskas, R., Fergusson, E., Brown, J., Poulton, N., Tupper, B., Labonté, J. M., et al. (n.d.). Improved whole genome amplification and the integrated study of genomic and optical properties of individual cells and viruses. *Manuscript in Preparation*.

Wang, Q., Garrity, G. M., Tiedje, J. M., & Cole, J. R. (2007). Naive Bayesian Classifier for Rapid Assignment of rRNA Sequences into the New Bacterial Taxonomy. *Applied and Environmental Microbiology*, *73*(16), 5261–5267. http://doi.org/10.1128/AEM.00062-07
