# Supplementary figures and images for: Strategies to improve reference databases for soil microbiomes
Source: ISME J. 2016 Dec 9;11(4):829–34. doi: 10.1038/ismej.2016.168 (PMC5364351; doi:10.1038/ismej.2016.168)

A

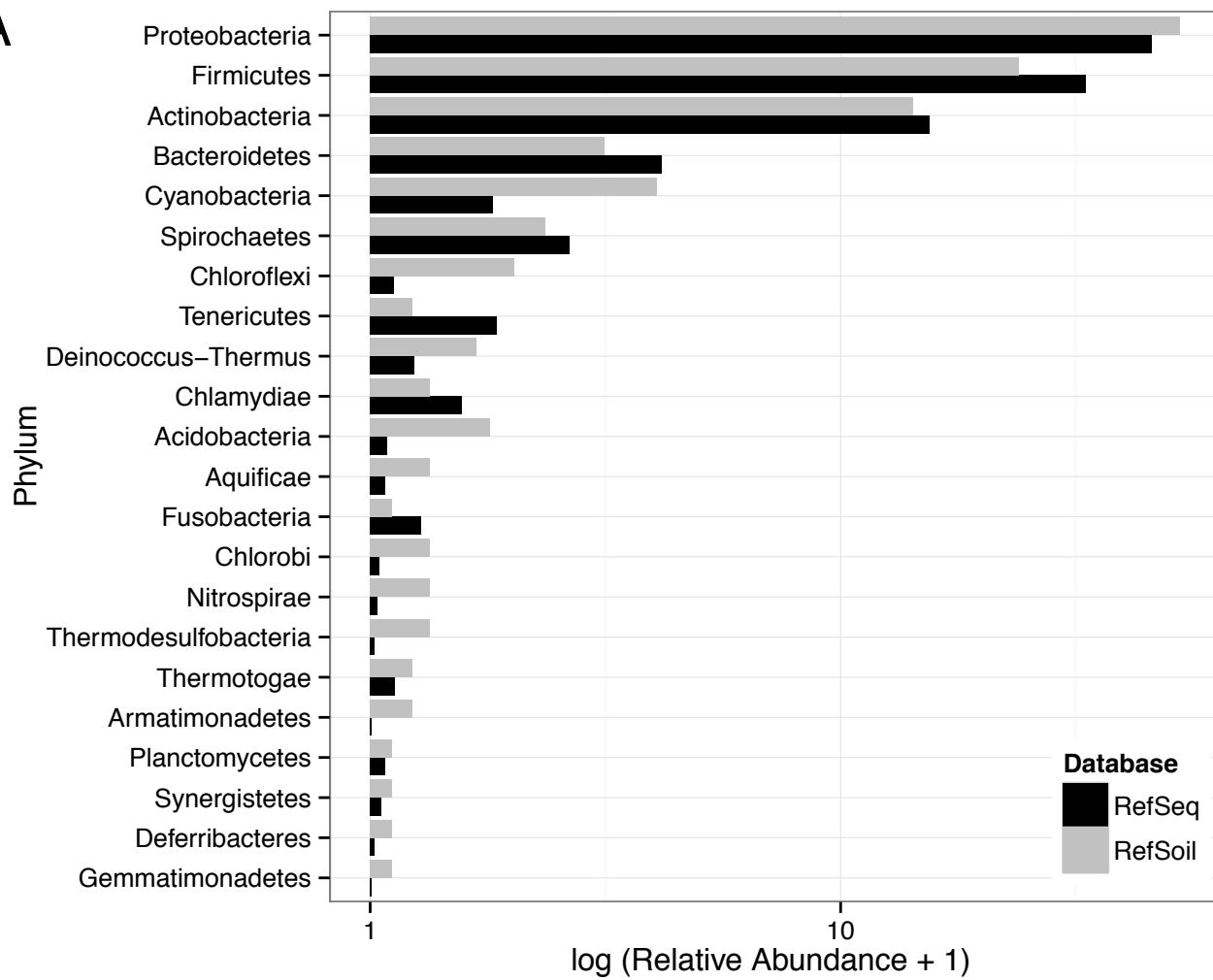

B

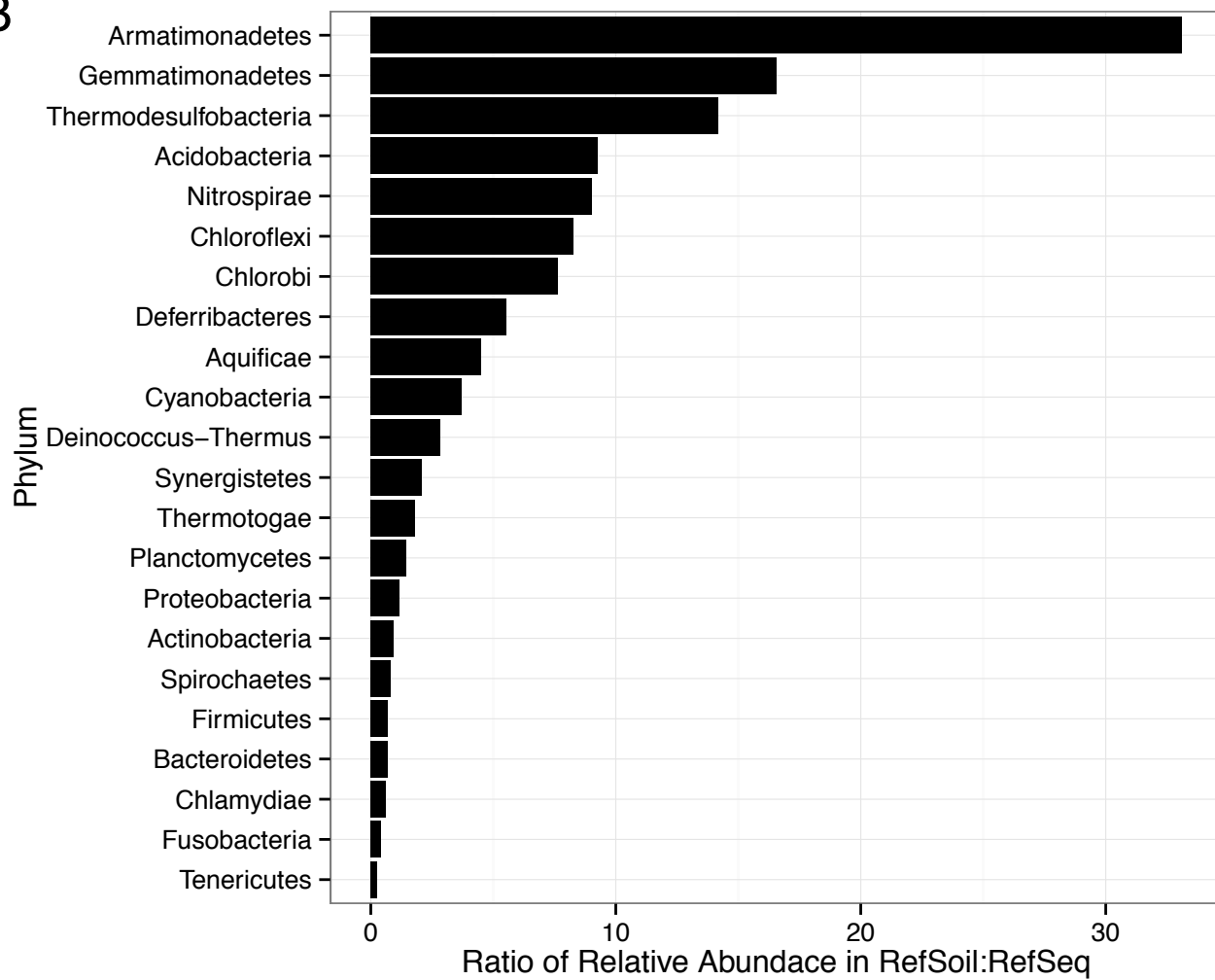

Supplement: Supplementary Figure 1 [file ismej2016168x2.pdf]

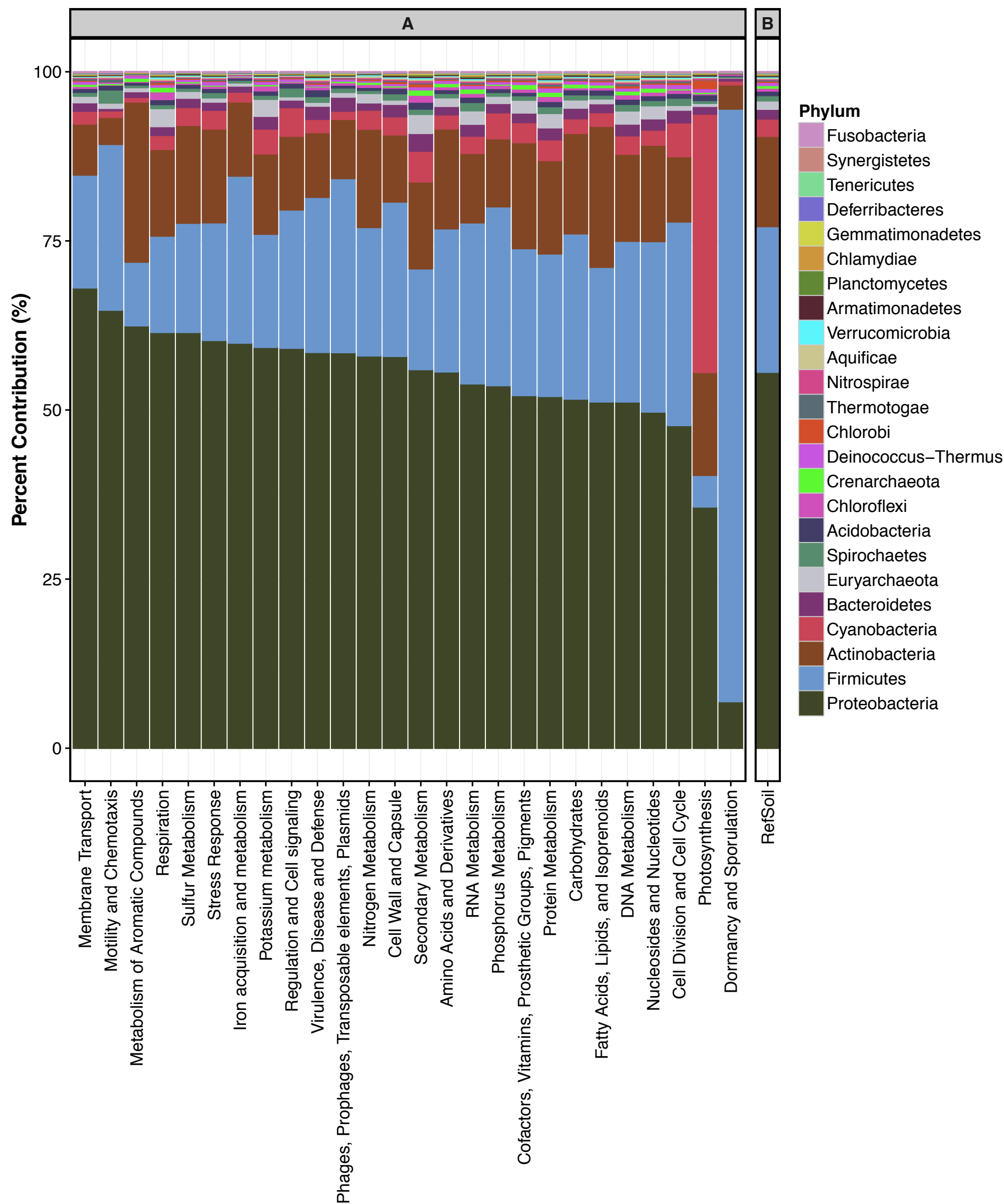

Supplement: Supplementary Figure 2 [file ismej2016168x3.pdf]

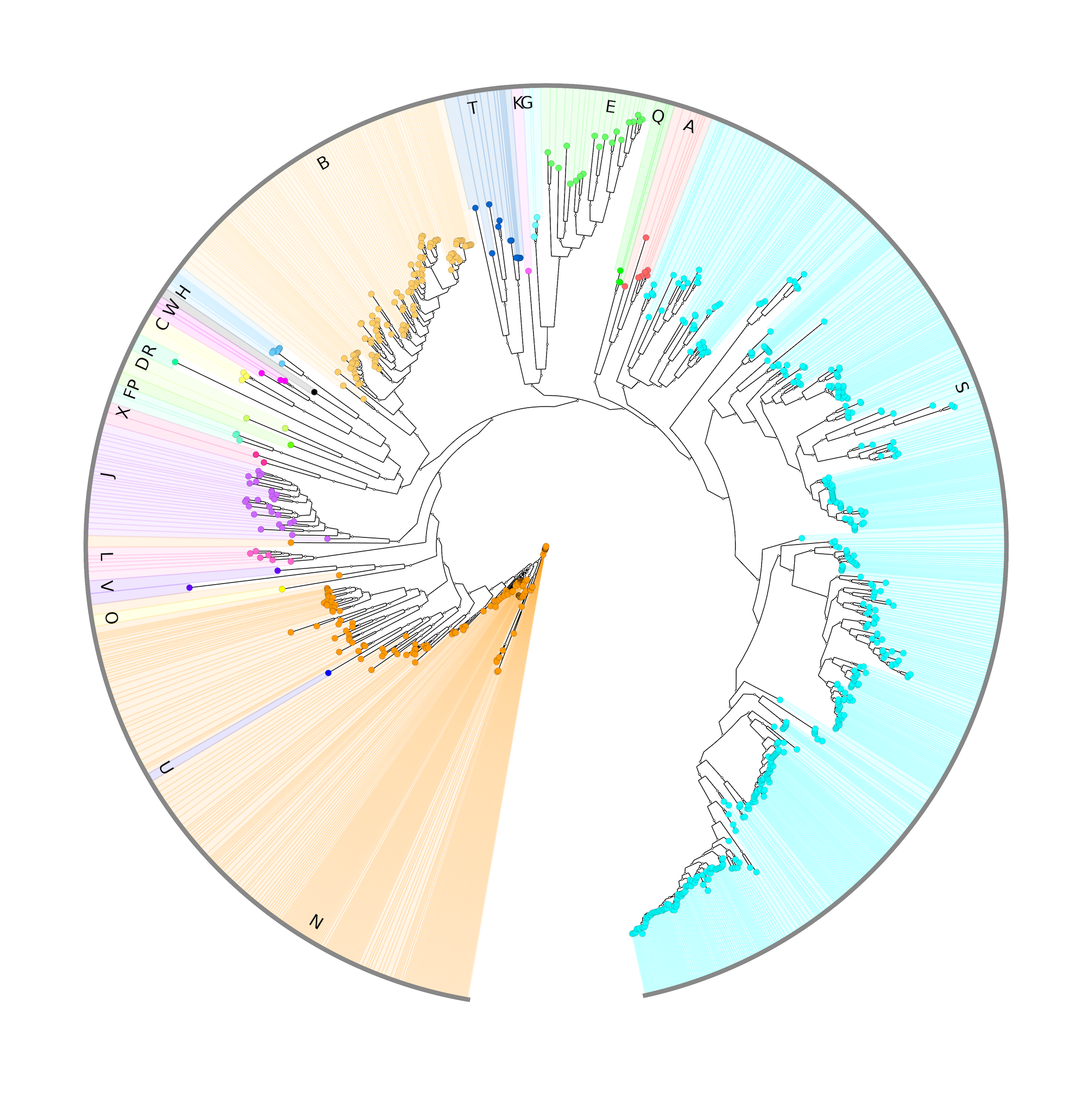

Supplement: Supplementary Figure 3 [file ismej2016168x4.png]

# Color Key

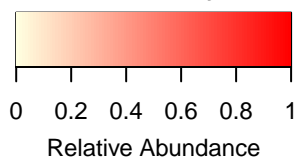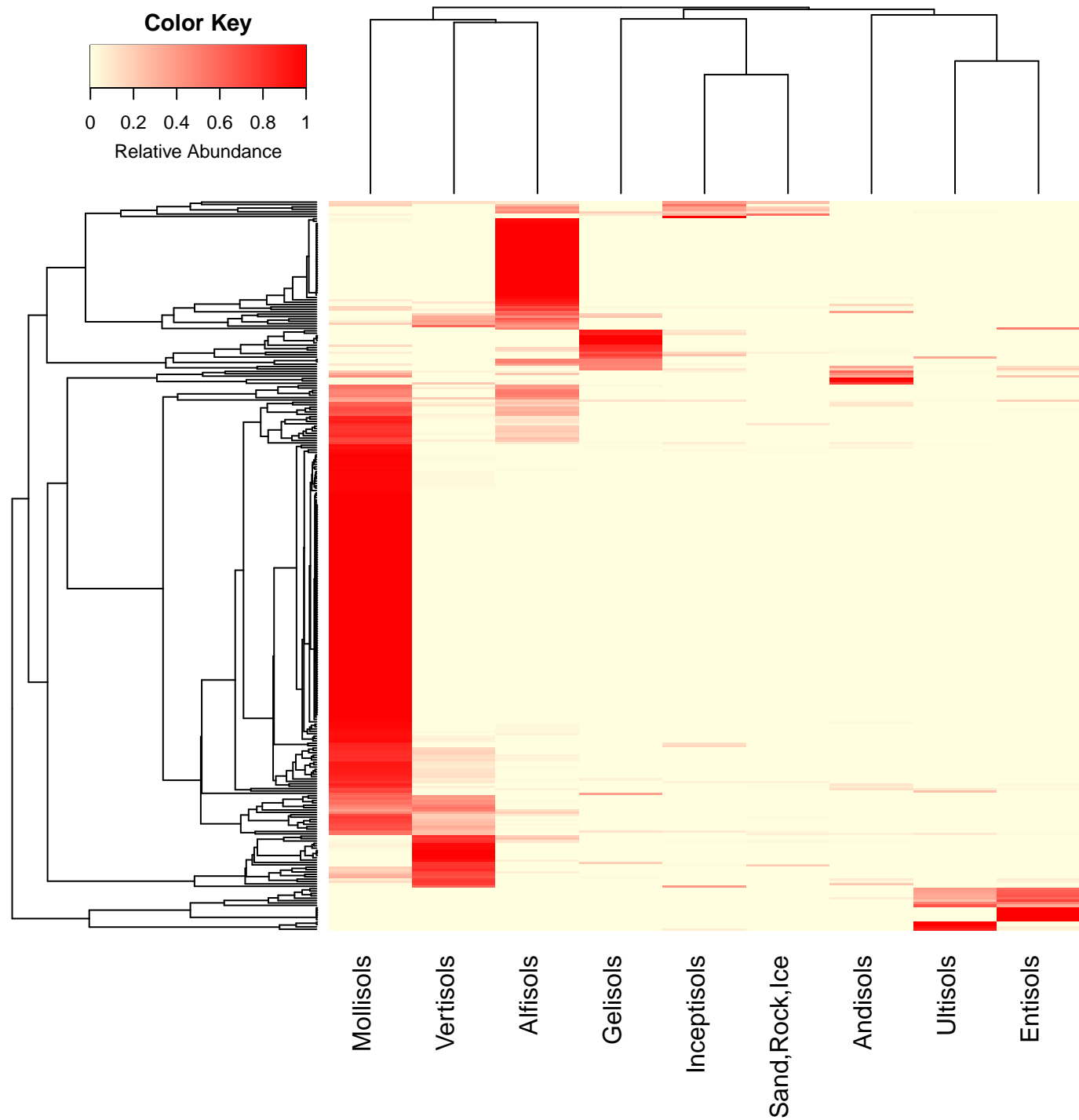

Supplement: Supplementary Figure 4 [file ismej2016168x5.pdf]
